# Supplementary material for: Resolving the full spectrum of human genome variation using Linked-Reads
Source: Genome Res. 2019 Apr;29(4):635–45. doi: 10.1101/gr.234443.118 (PMC6442396; doi:10.1101/gr.234443.118)
Supplement: Supplemental Material [file supp_gr.234443.118_Supplemental_longranger-2.2.zip › longranger-2.2.tar.bz2!/lr2.2-public/lib/stan/cmdstan/stan/doc/api/html/sub__row_8hpp.html]

Stan: src/stan/math/matrix/sub\_row.hpp File Reference


|  |  |
| --- | --- |
|  | Stan  2.5.0  probability, sampling & optimization |


- Main Page
- Related Pages
- Namespaces
- Classes
- Files

- File List
- File Members

All Classes Namespaces Files Functions Variables Typedefs Enumerations Enumerator Friends Macros Pages

- src
- stan
- math
- matrix

Namespaces |
Functions

sub\_row.hpp File Reference

`#include <stan/math/matrix/Eigen.hpp>`  
`#include <stan/math/error_handling/matrix/check_row_index.hpp>`  
`#include <stan/math/error_handling/matrix/check_column_index.hpp>`

Go to the source code of this file.

|  |  |
| --- | --- |
| Namespaces | |
|  | stan |
|  | Probability, optimization and sampling library. |
|  | |
|  | stan::math |
|  | Matrices and templated mathematical functions. |
|  | |

|  |  |
| --- | --- |
| Functions | |
| template<typename T > | |
| Eigen::Matrix< T,   1, Eigen::Dynamic > | stan::math::sub\_row (const Eigen::Matrix< T, Eigen::Dynamic, Eigen::Dynamic > &m, size\_t i, size\_t j, size\_t ncols) |
|  | Return a 1 x nrows subrow starting at (i-1,j-1). More... |
|  | |


---

[ Stan Home Page ]

*© 2011–2014,
Stan Development Team.*
